# Altered Metabolic Profile and Adipocyte Insulin Resistance Mark Severe Liver Fibrosis in Patients with Chronic Liver Disease

**DOI:** 10.3390/ijms20246333

**Published:** 2019-12-16

**Authors:** Melania Gaggini, Fabrizia Carli, Chiara Rosso, Ramy Younes, Romina D’Aurizio, Elisabetta Bugianesi, Amalia Gastaldelli

**Affiliations:** 1 Cardiometabolic Risk Unit, Institute of Clinical Physiology, CNR, 561214 Pisa, Italy; mgaggini@ifc.cnr.it (M.G.); fcarli@ifc.cnr.it (F.C.); 2 Division of Gastroenterology and Hepatology and Lab. of Diabetology, Department of Medical Sciences, University of Turin, 10124 Turin, Italy; chiara.rosso@unito.it (C.R.); ramy.younes@unito.it (R.Y.); ebugianesi@yahoo.it (E.B.); 3 Institute of Informatics and Telematics, CNR, 561214 Pisa, Italy; romina.daurizio@gmail.com

**Keywords:** lipidomics, metabolomics, insulin resistance, fibrosis, amino acids

## Abstract

Metabolomics/lipidomics are important tools to identify novel biomarkers associated with liver damage. Patients with chronic liver disease (CLD) and hepatitis C virus (HCV) infection often have alterations in glucose, lipid and protein metabolism. The aim of this study was to evaluate if dysfunctional lipid and amino acid metabolism was associated with fibrosis severity and insulin resistance in CLD/HCV patients. We analyzed the baseline sera of 75 subjects with CLD/HCV infection HCV genotype-1, with proven liver biopsy prior to antiviral treatment. We measured amino acid (AA) and lipid concentration by gas and liquid chromatography-mass spectrometry respectively. Alterations in peripheral glucose metabolism due to insulin resistance (IR) were assesed by HOMA-IR (Glucose x Insulin/22.5), while adipose tissue IR was estimated as (Adipo-IR = Free Fatty Acids x Insulin). Baseline HOMA-IR and Adipo-IR were related to the degree of liver fibrosis. Reduction in ceramides 18:1/22:0, 18:1/24:0, diacylglycerol 42:6 and increased phosphocholine 40:6 were associated with higher fibrosis. Adipo-IR was related to lower levels of lysophosphatidylcholine 14:0 and 18:2 and with higher levels of sphingomyelin 18:2/24:0 and 18:2/24:1. Almost all AA were positively associated with Adipo-IR but not with HOMA-IR. We further confirmed the potential use of metabolomics and lipidomics in CLD/HCV subjects finding novel biomarkers of hepatic fibrosis and show that the adipose tissue IR is associated with more severe liver disease and is an important marker not only of altered lipid but also AA metabolism.

## 1. Introduction

Metabolomics and lipidomics are used to assess the metabolic factors involved in the onset of metabolic diseases, to understand the different metabolic pathways involved in organ damage [1,2]. Several studies have shown that in patients with chronic liver disease (CLD) and HCV infection, insulin resistance when present affects the liver, muscle and adipose tissue by altering both glucose and lipid homeostasis [3,4]. Chronic hepatitis C (CHC) infection leads to hepatic inflammation, which stimulates liver fibrosis [5]. Moreover, HCV infection might be able to disrupt glucose and lipid metabolism via up-regulation of TNF-alfa and/or down-regulation of suppressors of cytokine signaling and protein phosphatase 2A [6] that could be implicated in hepatic and extra-hepatic IR [4]. These metabolic derangements had been associated with the progression of liver fibrosis and significantly contributed to a decrease in sustained virological response rate [7] and might persist after completion of treatment.

Liver fibrosis is due to excessive accumulation of extracellular matrix proteins, including collagen [8], in response to tissue damage caused by HCV infection. Although recent advancements in direct-acting antiviral (DAA) treatment are able to eliminate viremia, very few treatment strategies can effective prevent the advancement of liver fibrosis in these patients [5]. Several lipid species, including mono- (MAGs), di- (DAGs) and tri-acylglycerol (TAGs), ceramides (CER) and lysophosphatidylcholines (LPC) are implicated in chronic inflammation with consequent effects on multiple organs [9]. In particular the high content of long chain saturated fatty acids (SFA), both in serum and organs, is associated with lipotoxicity and a worse outcome. Lipotoxicity is the result of increased peripheral lipolysis due to adipose tissue IR with overflow of free fatty acids to the liver that are in part stored as triglycerides (steatosis) or converted to lipotoxic species like ceramides [10], although impaired suppression of lipolysis by insulin have been shown by some [11], but not others [4] in patients with HCV. Previous studies in patients with NAFLD have associated increased adipose tissue IR to more severe liver fibrosis [12,13,14], and recently, Lim et al. have confirmed these data in patients with CHC [11].

Not only lipid, but also amino acid (AA) metabolism is altered in patients with chronic liver disease. The serum levels of branched-chain amino acids (BCAA, i.e., valine, leucine and isoleucine) are usually decreased in patients with liver cirrhosis, while aromatic amino acids (AAAs), such as tyrosine and phenylalanine, are increased [15]. We have recently shown that AA concentrations, in particular BCAA, glutamate, serine and glycine, vary with the degree of liver fibrosis in NAFLD [16]. Furthermore, a novel index derived from the AA implicated in the synthesis of glutathione (glutamate, serine and glycine) and ceramides (i.e., serine), which we called GSG index, was higher in subjects with severe fibrosis (F3-F4) compared to those with mild/moderate fibrosis and increased proportionally to IR in both liver and adipose tissue [16].

Considering these previous results, the aim of our study was to evaluate in CLD/HCV patients if dysfunctional lipid and amino acid metabolism was associated with fibrosis severity and insulin resistance by measuring alterations in metabolomic/lipidomic profile.

## 2. Results

The clinical characteristics of the patients divided for fibrosis stage are shown in Table 1. Subjects with more severe fibrosis were slightly older but, except for GGT (higher in subjects with severe fibrosis, i.e., F5–F6, *p* = 0.02), all clinical variables were similar.

### 2.1. Markers of Insulin Resistance and Inflammation in CLD

Initially, we focused our attention on the associations between degree of liver fibrosis (as an index of severity of CLD) and IR indexes, i.e., HOMA, which reflects impaired insulin action in glucose metabolism, and Adipo-IR, which reflects the impairment of the antilipolytic effect of insulin resulting in higher circulating free fatty acids. Subjects were grouped according to the severity of liver fibrosis as: low (F1–F2), mild (F3–F4) or severe (F5–F6) fibrosis. We found that not only HOMA-IR, but also Adipo-IR was significantly increased with the worsening of fibrosis (Table 1). The leptin and MCP-1 serum concentration were similar among patients with different stages of fibrosis or IR (Table 1), although leptin was associated with SVR.

### 2.2. Lipidomic Profile in Relation to Fibrosis Stage and Insulin Resistance

**Figure 1:**
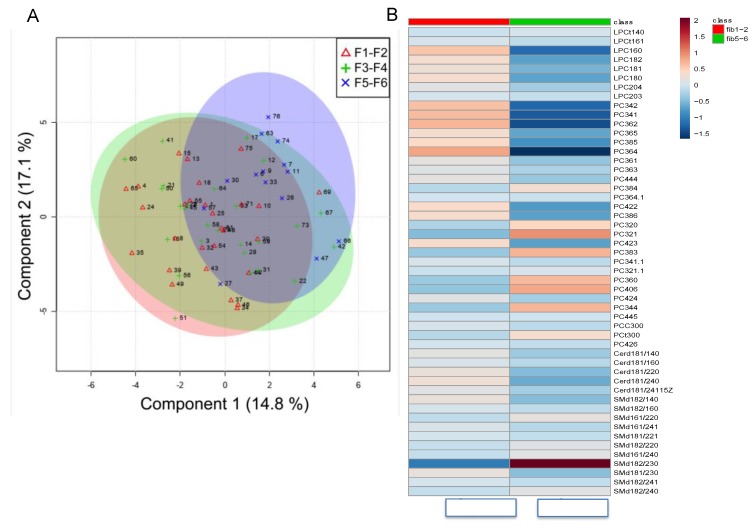
(**A**) Scores plot of first two PLS components obtained by Partial Least Squares-Discriminant Analysis (PLS-DA). (**B**) Heat maps of plasma lipid according to low (F1–F2) and severe (F5–F6) fibrosis level.

**Figure 2:**
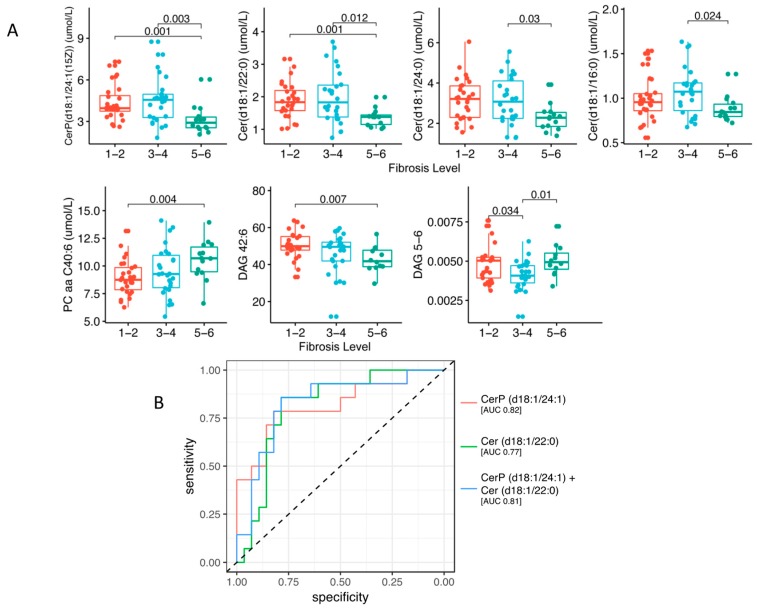
(**A**) Box plot with metabolite levels of single samples, group mean and quartiles. Kruskal-Wallis *p*-values < 0.05 are shown. In red Fibrosis F1–F2; in light blue Fibrosis F3–F4; in green Fibrosis F5–F6 (**B**) ROC for the best two metabolites in discriminating between low (F1–F2) and severe (F5–F6) fibrosis level.

**Figure 3:**
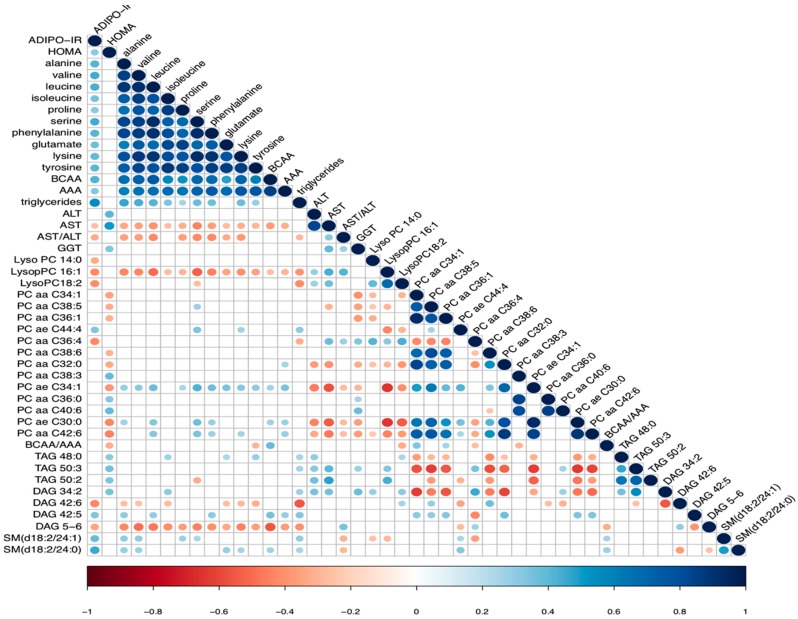
Correlation matrix with a selection of lipid classes and amino acids that were positively/negatively correlated to Adipo-IR and HOMA-IR. Only correlations with absolute Spearman rho > 0.3 (gradient color scale) and *p*-value < 0.05 are shown.

We performed targeted lipidomics of sera by ultra-high performance liquid chromatography-quadrupole time-of-flight mass spectrometry (UHPLC/QTOF-MS). The lipid composition and concentrations of the following species were measured: ceramides (CER), phosphocholines and lyso-phosphocholines (PC, LysoPC) for which we report both quantitative and qualitative analyses, mono-, di- and tri-acylglycerols (MAGs, DAGs, TAGs) and sphingomyelin (SM), which were evaluated only in qualitative mode. Lipidomic profiles were globally compared by using Partial Least Square Discriminant Analysis (PLDA), a supervised classification method which optimizes the separation among the different groups of samples (Figure 1A). PLDA shows that subjects with severe fibrosis (F5–F6) are separated by the other two groups. Heat maps reveal that several species of lipids were related to the increase of fibrosis stage (Figure 1B). However, seven metabolites were differentially abundant among the three groups (Figure 2A). Specifically, PC 40:6 was higher in subjects with fibrosis grade F5–F6 versus F1–F2 (*p* = 0.004), while ceramides CER(18:1/24:1), CER(18:1/22:0), CER(18:1/24:0) and CER(18:1/16:0) generally showed lower concentrations in F5–F6 respect to F1–F2 or F3–F4 (Kruskal-Wallis *p* < 0.05, see Figure 2A for details). Moreover, concentrations of DAG 42:6 e in general DAG with more than five double bounds (DAG 5–6, i.e., containing mainly unsaturated fatty acids) were different among the three groups. Using binomial logistic regression analysis to discriminate between extreme groups (low versus severe fibrosis) showed that ceramides CER(18:1/24:1) and CER(18:1/22:0) (as single variables or in combination) are the best ceramides species able to discriminate the severity of fibrosis as measured by ROC-AUC (Area Under the ROC) (Figure 2B). Moreover, several lipids were associated with IR either in the adipose tissue or muscle (Figure 3).

### 2.3. Association between Amino Acid Profile, Fibrosis Stage and Insulin Resistance

AA concentrations were measured in serum of all patients by GC/MS and related to IR and fibrosis stage. Almost all AA were positively associated with the presence of IR, in particular with Adipo-IR but not with HOMA-IR (Figure 3).

**Figure 4:**
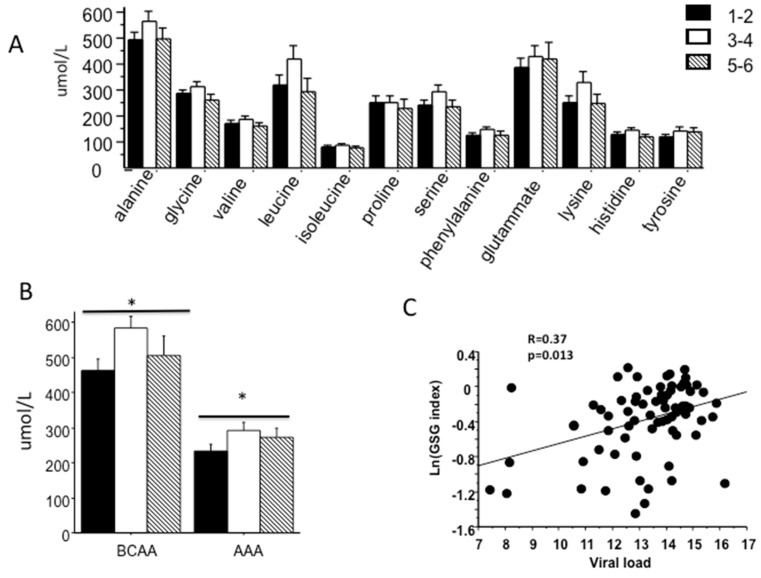
Basal amino acid profile (**A**), branched chain (BCAA) and aromatic (AAA) amino acids concentrations (**B**), according to fibrosis stage. * *p* value <0.05. Association between GSG index and Viral load (**C**)

Only the combination of BCAA and AAAs were increased with fibrosis (*p* = 0.023; Figure 4B), while the BCAA/AAA ratio was not related to fibrosis stage. The GSG index (i.e., the ratio of glutamate/(serine and glycine)), that combines AA involved in glutathione and ceramides synthesis, was significantly associated with increased liver enzymes (GGT, R= 0.37 *p* = 0.0027 and AST, R = 0.46 *p* = 0.022) confirming our previous report showing the association of GSG index with liver damage [16]. GSG index tended to be higher in patients with fibrosis stage ≥ 5 (Figure 4C) and was associated with viral load.

### 2.4. Baseline Markers of Response to Therapy (SVR)

Our secondary aim was to evaluate if any of the lipids or amino acids measured pre-treatment could identify patients with a better sustained virological response to treatment. The 75 patients were divided in two groups, i.e., “responders” when a sustained virological response (SVR) was obtained (*n* = 36 patients, coded as “R”) versus “non responders” (*n* = 39 patients, coded as “NR”)

First, we observed that pre-treatment degree of IR was associated with SVR as both HOMA-IR and Adipo-IR were higher at baseline in “NR” subjects compared to “R” (HOMA-IR 48.4 ± 4.3 versus 36.8 ± 3.1; Adipo-IR 16.9 ± 2 versus 12.3 ± 1.2).

**Figure 5:**
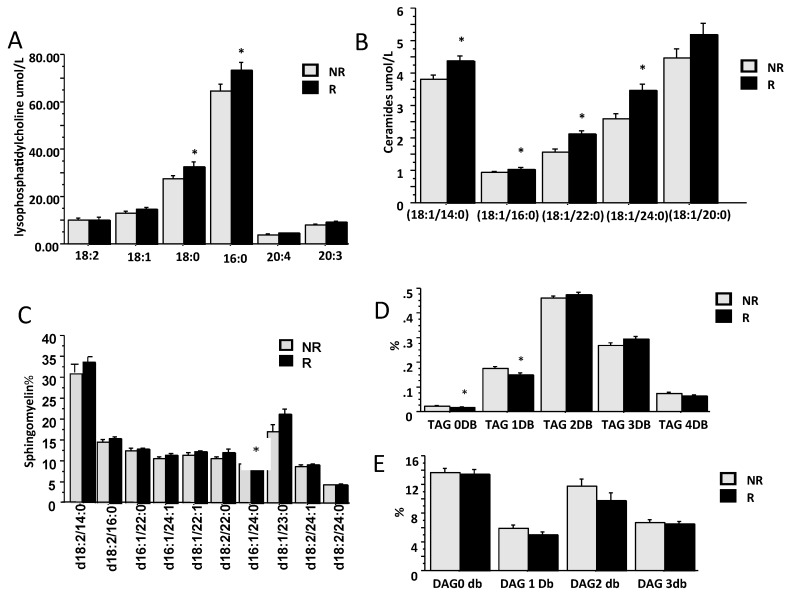
Basal lipid composition in responders (R) and non responders (NR): serum concentration of LysoPC (**A**), ceramides (**B**) and sphingomyelin (**C**); degree of desaturation (number of double bonds, DB) of TAG (**D**) and DAG (**E**).

Second, lipidomic analysis showed that “R” patients showed increased concentrations of LysoPC, in particular saturated LysoPC like LPC 16:0 and 18:0 (*p* < 0.05) and ceramides (*p* = 0.0006), in particular of CER(18:1/14:0), CER(18:1/16:0), CER(18:1/22:0) and CER(18:1/24:0). Among sphingomyelin (SM), only SM 16:1/24:0 was higher in “R” compare to “NR” while no difference was observed in PC profile. The analysis of TAGs and DAGs profile showed similar serum concentrations total TAGs and DAGS although patients “NR” have more saturated TAGs compared to “R” patients (*p* < 0.05), while no difference was observed in DAGs between the two groups (Figure 5)

**Figure 6:**
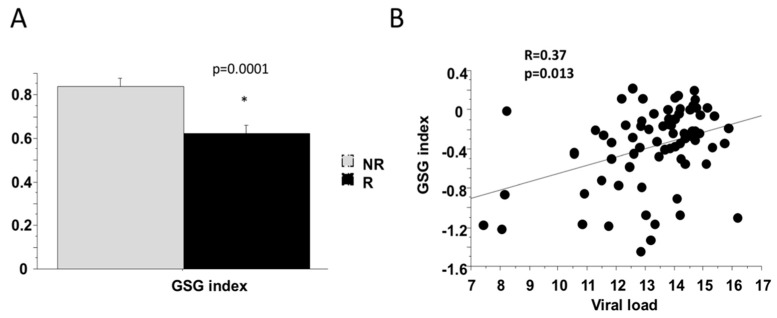
Basal GSG index was higher in NR patients (**A**) and positively associated with viral load (**B**). * *p* value= 0.05.

The amino acid profile was different between “R” and “NR” patients. “NR” patients had higher serum concentrations of glutamate and proline than patients responding to PEG-IFN therapy, while the concentrations of other amino acids, including AAA and BCAA were similar in the two groups (Table 2). Several AA were related to viral load such as glutamate, isoleucine, lysine, proline, tyrosine BCAA and AAA. We founded that GSG index was associated with viral load and “NR” patients had significantly higher GSG index before treatment compared to “R” patients (Figure 6).

## 3. Discussion

Several studies have used lipidomic and metabolomic analyses of serum samples to identify early biomarkers of liver disease in NAFLD/NASH, while only a few have used these novel tools to identify biomarkers of liver damage (particularly fibrosis stage) and response to treatment in patients with chronic liver disease of a different etiology. In patient with liver disease of varying etiology and severity, insulin resistance and amino acid metabolism are altered, in particular AAA, which is metabolized mainly by the liver [17]. Moreover, HCV infection is associated with alterations in glucose, lipid and amino acid metabolism, lipid synthesis and clearance and insulin resistance [4,18]. Hepatitis C virus consist of replicates principally in hepatocytes; other organs are also affected by the virus, such as muscle, adipose tissue and pancreas [11,19,20], leading to comorbidities such as type 2 diabetes, renal and cardiovascular disease and fibrosis in liver [5].

We measured the lipidomic and metabolomic profiles and their association with insulin resistance in relation to liver fibrosis and virological response to treatment in patients with chronic liver disease and HCV infection. Insulin resistance to glucose and lipid metabolism is a pro-fibrogenetic stimulus [21]. Previously, researchers has shown that patients with HCV have impaired glucose metabolism due to increased IR in both muscle and liver, and this was associated with more severe liver fibrosis [3,4,22]. IR may be present also in adipose tissue due to the resistance to the antilipolytic effect of insulin resulting in increased lipolysis that in turn promotes liver lipotoxicity, as a consequence of FFA overflow to the liver [10]. In this study, our patients showed IR both in liver/muscle and at the level of adipose tissue, and this was associated with the worsening of fibrosis, in agreement with recent data by Lim et al. [11]. In subjects with NAFLD adipose tissue, IR is associated with the severity of hepatic fibrosis [12,13,14]. However, while most of patients with NAFLD have excess fat accumulation, most of the CLD patients are lean; thus, increased Adipo-IR might explain the low subcutaneous fat accumulation due to accelerated lipolysis.

The adipose tissue is also an endocrine organ that secretes hormone such as leptin, which plays an important role in the regulation of body fat and in the development of hepatic fibrosis [23]. In our cohort of patients, leptin was higher in “NR” patients before treatment, although it was not associated with IR, fibrosis staging or steatosis, similar to the results obtained by Grasso et al. [24]. High baseline serum leptin levels might represent a negative prognostic factor for response to antiviral therapy that should be further investigated.

We studied if and how the lipidomic profile was associated with fibrosis stage (i.e., low (F1–F2), mild (F3–F4) or severe (F5–F6)). We found that seven serum lipid species resulted differentially abundant among the three groups. Particularly PC 40:6 was higher in F5–6 versus F1–2, while ceramides CER (18:1/24:1), CER (18:1/22:0), CER (18:1/24:0) and CER (18:1/16:0) showed lower levels in F5–6 respect to F1–2 or F3–4. Moreover, DAG with more than five double bounds (DAG 5–6) and DAG (42:6) were found different among the three groups of subjects. The logistic regression analysis used to discriminate between extreme fibrosis groups (i.e., low versus severe) showed that ceramides CER (18:1/24:1) and CER (18:1/22:0) (as single variables or in combination) were the best in discriminate severity of fibrosis (Figure 2B). Ceramides have been identified as potent regulators of signaling in several biological pathways and affect hepatocellular susceptibility to various stimuli [25] including the regulation of HCV replication [26]. Ceramide molecules are composed of a sphingosine backbone bound to fatty acids with different chain lengths. Recent studies have revealed that long-chain (C16–C20) and very-long-chain (C22–C24) ceramides express opposite effects on cell proliferation, i.e., very long chain CER promote proliferation in vitro contrary to the pro-apoptotic effects of long chain CER, in various cell types [27,28,29]. In our study, the serum concentration of two very long chain ceramides (CER (18:1/24:1) and CER (18:1/22:0)) was lower in severe fibrosis. Moreover, patients with severe liver fibrosis had higher levels of PC 40:6.

We also observed differences in phospholipids abundances, particularly between responder and non-responder, and several phospholipids were associated with IR as shown by the matrix correlation in Figure 3. This result agrees with previous data that reported that phospholipids play a critical role in physiological processes and in modulation of insulin sensitivity [30].

Not only lipid, but also amino acid metabolism is frequently altered in patients with liver diseases [31]. An imbalance in the levels of AAAs or BCAAs is commonly seen in patients with chronic liver disease [17] and most changes in serum AA concentrations in patients with CLD could be due to the impairment in hepatic function, hyperinsulinemia and hyperglucagonemia [32]. In the present study, our results showed that only BCAA and AAAs were associated with severity of fibrosis. Particularly the amino acid profile has highlighted that almost all AA were associated with the increase of adipose IR and not with muscle IR. Our previous data in non-obese NAFLD have shown that alteration in AA metabolism is independent of degree of obesity, but associated with IR. We have found higher serum levels of isoleucine and valine (BCAAs), tyrosine (AAA), alanine, lysine and glutamate in subjects with fatty liver disease while the plasma concentration of glycine was significantly lower; the GSG index, (glutamate/serine + glycine) was related to an increased level of liver enzymes, particularly gamma-glutamyltransferase, and liver fibrosis in non-obese and obese NAFLD [16]. In this group of patients we also found that GSG index was significantly associated with liver enzymes and tended to be higher in patients with severe fibrosis. The increase in GSG index reflects an alteration in hepatic function and/or related to an increased demand in glutathione (GSH) and its transamination by GGT, confirming our previous report that showed the association between GSG index and liver damage in patients with NASH [16].

The secondary aim of this study was to identify which metabolite/lipid was associated with viral load and SVR. Several amino acids were related to baseline viral load i.e., glycine, isoleucine, lysine, proline tyrosine and AAAs. Patients who got SVR had significant lower baseline serum concentrations of glutamate and proline and the GSG index. Lipidomic analysis showed that several classes of lipid were higher in serum of patients “NR” before treatment. We observed that ceramides CER (18:1/14:0), CER (18:1/22:0), CER (18:1/24:0) and Lyso-PCs LPC-18:0 and LPC-16:0 were different in (R) patients compare (NR). We also found that degree of fatty acid unsaturation, evaluated by number of double bonds in TAGs and DAGs, was higher in patients “NR” (Figure 5). This negative metabolic profile in “NR” patients indicates the importance of lipid glucose metabolism in all CLD.

This study has some limitations. First it is a cross-sectional study, making it difficult to dissect the relationship between the new biomarkers discovered and their interplay with the metabolic complications and liver disease/severity. Although the relationship between baseline metabolic alterations and response to treatment were significant, the treatment here used as an example of SVR is no longer used. Thus, these findings should be investigated after the current most common treatment, DAAs, to confirm if metabolic factors are crucial for successful response to treatment. Moreover, a group of subjects with liver disease (i.e., a control group) would be useful to better understand the metabolic differences in patients with different chronic liver diseases.

In conclusion, this study confirmed that Adipo-IR is associated with more severe liver disease also in patients with CLD/HCV and is an important marker not only of altered lipid but also AA metabolism. Moreover, the GSG index was associated to high viral load before treatment. These results further confirmed the use of metabolomics and lipidomics for the identification of subjects with most severe liver disease.

## 4. Materials and Methods

We performed metabolomic/lipidomic analyses on the sera of 75 patients positive for HCV (all with Genotype-1) previously enrolled in the Gastro-Hepatology Division of the University Hospital Torino, Italy for a multicenter project (described in reference [33]). Inclusion criteria were: (1) diagnosis of chronic hepatitis C with G1 infection based on hepatitis C serology and viral RNA, (2) histologic diagnosis on liver biopsy, and (3) alcohol consumption less than 20 g/d in the past 12 months. All participants were recruited after providing written informed consent. Some clinical and biochemical characteristics of the study cohort has been previously published [33]. Patients were excluded from the study if they were co-infected with either the hepatitis B virus or human immunodeficiency virus, if they were affected from diabetes or if they were not of northern European descent. All patients were tested at the time of biopsy for HCV RNA (limit of detection 12 IU/mL). HCV RNA levels were quantified at baseline and viral load was classified as low (≤850,000 IU/mL) or high (≥850,000 IU/mL).

Primary aim of this study was to evaluate which metabolites (AA or lipids) were altered in patients with CLD and severe fibrosis. Liver fibrosis was staged in the biopsy according to an Ishak score [34]. For this analysis, patients were divided in three groups according to the severity of liver fibrosis, according to the Ishak score [34]: as low (F1–F2), mild (F3–F4) or severe (F5–F6) fibrosis. Low fibrosis F1–F2: fibrous expansion of some portal areas, with or without short fibrous septa (score 1), fibrous expansion of most portal areas, with or without short fibrous septa (score 2). Mild fibrosis F2–F3: fibrous expansion of most portal areas with occasional portal to portal (P-P) bridging (score 3), fibrous expansion of portal areas with marked bridging portal to portal (P-P) as well as portal to central (P–C)) (score 4). Fibrosis severe F5–F6: marked bridging (P-P and/or P-C) with occasional nodules (incomplete cirrhosis) (score 5), cirrhosis, probable or definite (score 6). The metabolomic/lipidomic analyses were performed by mass spectrometry (see below). In addition, we measured markers of adipose tissue dysfunction, as serum concentration of leptin and monocyte chemoattractant protein-1 (MCP-1), a marker of microphage inflammation by Luminex technology (Merckgroup, Darmstadt, Germany), and free fatty acid (FFA) concentrations, (Fujifilm WAKO Diagnostic, USA). Liver enzymes were measured by chemistry analyzer Beckman Coulter Olympus AU400 (Ireland).

A secondary aim was to evaluate which metabolite/lipid was associated with sustained virological response (SVR). In this group, 36 patients responded to the antiviral therapy (R), based on SVR after treatment with PEG-IFNa plus RBV, while 39 patients were considered as non-responders (NR).

### 4.1. Lipidomic and Metabolomic Measurements

Lipidomic analysis was performed using a High Performance Liquid Chromatography (Agilent UHPLC 1290) coupled with a Quadrupole Time-Of-Flight Mass Spectrometry QTOF (QTOF-MS, Agilent 6540) equipped with electrospray ionization source (ESI). Fasting serum samples (10 uL) were first deproteinized using cold methanol (200 uL). For liquid chromatography analysis, we used an Agilent ZORBAX Eclipse Plus C18 2.1 × 100 mm 1.8-Micron column at 50 °C. The mobile phase A was water with 0.1% formic acid and the mobile phase B was isopropanol/acetonitrile (1:1, *v*:*v*) with 0.1% formic acid. Injection volume was 1 uL. We performed untarget acquisition of sample spectra. Quantitative analysis was performed with internal standards added to the sample before deproteinization, i.e., Ceramide (CER (d18:1/17:0)), Phosphocholine (PC(17:0/17:0)) and Lysophosphatidylcholine (LPC(17:0)) (Avanti Polar Lipids, Sigma Aldrich, St Louis, MO, USA). For MAGs, DAGs, TAGs and sphingomyelin (SM), we performed a qualitative analysis and evaluated the proportion of unsaturated to saturated fat (i.e., double bonds).

Metabolomic analysis (polar metabolites) was performed by Gas Chromatography (GC)-Mass Spectrometry (MS) (Agilent technology GC-7890/MS-5975; Santa Clara, CA, USA), equipped with a DB-5 column (J&W, Agilent, Santa Clara CA, USA). Briefly, 80 uL of serum sample were deproteinized with 1 mL of cold methanol after adding a mix of ^13^C labeled internal standards of amino acids (Celtone, CIL Cambridge, MA, USA) that contains uniformly labeled phenylalanine, tyrosine, leucine, isoleucine, valine; alanine, lysine, hystidine, proline, threonine, glutamate, serine and glycine. After deproteinization, samples were purified through a cationic resin (BIORAD), dried under nitrogen, derivatized using N-methyl-N-(tert-butyldimethylsilyl) trifluoroacetamide (MSTBSTFA, Sigma, USA) and injected in the GCMS equipped with an Agilent J&W HP-5ms column. The spectra were acquired in SIM/SCAN mode.

### 4.2. Calculation and Statistical Analysis

We calculated indexes of insulin resistance as HOMA-IR index (fasting glucose × fasting insulin/22.5) that reflects fasting insulin resistance relative to glucose metabolism in muscle/liver [35] and adipose tissue IR (Adipo-IR) index evaluated as the product (FFA × insulin) [36], which measure the resistance to the antilipolytic effect of insulin.

From amino acids profile, we calculated Equations (1)–(3); Equations (1) includes AA involved in the synthesis of glutathione (glutamic acid and glycine) and of ceramides (serine) [16]:

GSG index = glutamic acid/(serine + glycine)(1)

BCAAs = (leucine + valine + isoleucine)(2)

AAAs = (tyrosine + phenylalanine)(3)

BCAA/AAA, which is reduced with severity of liver disease, mainly because the concentrations of AAA that are metabolized mainly in liver tend to be increased with severity of liver disease as a sign of the impairment in hepatic metabolism [17].

For DAG and TAG, we calculated the number of double bonds, i.e., the degree of unsaturation.

Metabolomic and lipidomic data were jointly analyzed using MetaboAnalystR 1.0.3 software (XiaLab at McGill University, Montreal, Quebec, Canada) [37]. After log transformation and auto scaling (e.g., mean-centered and divided by standard deviation of each variable), Principal Component Analysis (PCA), Partial Least Squares-Discriminant Analysis (PLS-DA) and heat maps were performed using MetaboAnalystR 1.0.3. We performed quality control of samples using PCA that allowed us to label the 68 samples as outliers, which were then excluded from downstream analysis. Nonparametric statistical methods, including Mann−Whitney U test, Kruskal−Wallis and Spearman rank correlation analysis, were performed using “R: A Language and Environment for Statistical Computing” (R Foundation for Statistical Computing, 2008 Vienna, Austria) or SPSS 13.0 (SPSS Inc, Chicago, IL, USA) as data were not normally distributed. Regression analysis and Receiver operating characteristic (ROC) curves for modulated single or combined metabolites were performed using the R software as a binary classification model for discriminating low from high level fibrosis and evaluated by the area under the curve (AUC) and 95% confidence interval, as well as *p*-value. Data are presented as the mean ± SEM.

## Figures and Tables

**Table 1:** Clinical characteristics of Chronic Liver disease/HCV patients according with fibrosis stage.

Variable	F1–F2	F3–F4	F5–F6	*p* Value
Gender (M/F)	15/14	11/15	6/6	ns
Age (y)	45.5 ± 2.14	48.3 ± 2.3	52.5 ± 3.1	0.026
BMI	23.2 ± 0.6	23.8 ± 0.6	24.7 ± 1.3	ns
Cholesterol (mg/dL)	169.2 ± 7	169.3 ± 8.4	161.1 ± 7.7	ns
LDL (mg/dL)	102.5 ± 4.9	101.4 ± 6.6	97.5 ± 6.8	ns
HDL (mg/dL)	41.9 ± 2.6	38.7 ± 3.7	39.5 ± 4.3	ns
Triglycerides(mg/dL)	123.7 ± 9.9	146.2 ± 17.5	120.6 ± 13	ns
AST (mU/L)	8.0 ± 0.9	6.2 ± 0.7	8.9 ± 1.3	ns
ALT (mU/L)	9.3 ± 1.1	7.7 ± 0.9	8.2 ± 1.13	ns
GGT (mU/L)	38 ± 3.6	31 ± 4.4	58.6 ± 10.5	0.021
Glucose (mg/dL)	67.8 ± 2.3	68.2 ± 3.2	76.4 ± 5.2	ns
Insulin ulU/mL	11.9 ± 0.9	12.8 ± 1.3	16.1 ± 1.8	ns
MCP-1 pg/mL	223.9 ± 16	218.8 ± 26.	188.4 ± 22.2	ns
Leptin pg/mL	3000 ± 632	3149 ± 547	65,340 ± 2345	ns
HOMA-IR	36.6 ± 1.2	38.7 ± 3.8	54.6 ± 6.3	0.024
Adipo-IR	10.7 ± 1.2	18.1 ± 2.4	18.8 ± 3.2	0.042
Gender (M/F)	15/14	11/15	6/6	ns
Age (y)	45.5 ± 2.14	48.3 ± 2.3	52.5 ± 3.1	0.026
BMI	23.2 ± 0.6	23.8 ± 0.6	24.7 ± 1.3	ns
Cholesterol (mg/dL)	169.2 ± 7	169.3 ± 8.4	161.1 ± 7.7	ns
LDL (mg/dL)	102.5 ± 4.9	101.4 ± 6.6	97.5 ± 6.8	ns
HDL (mg/dL)	41.9 ± 2.6	38.7 ± 3.7	39.5 ± 4.3	ns
Triglycerides(mg/dL)	123.7 ± 9.9	146.2 ± 17.5	120.6 ± 13	ns
AST (mU/L)	8.0 ± 0.9	6.2 ± 0.7	8.9 ± 1.3	ns
ALT (mU/L)	9.3 ± 1.1	7.7 ± 0.9	8.2 ± 1.13	ns
GGT (mU/L)	38 ± 3.6	31 ± 4.4	58.6 ± 10.5	0.021
Glucose (mg/dL)	67.8 ± 2.3	68.2 ± 3.2	76.4 ± 5.2	ns
Insulin ulU/mL	11.9 ± 0.9	12.8 ± 1.3	16.1 ± 1.8	ns
MCP-1 pg/mL	223.9 ± 16	218.8 ± 26.	188.4 ± 22.2	ns
Leptin pg/mL	3000 ± 632	3149 ± 547	65,340 ± 2345	ns
HOMA-IR	36.6 ± 1.2	38.7 ± 3.8	54.6 ± 6.3	0.024
Adipo-IR	10.7 ± 1.2	18.1 ± 2.4	18.8 ± 3.2	0.042

BMI = Body Mass Index; LDL = Low Density Lipoprotein; HDL = High Density Lipoprotein; AST = Aspartate Aminotransferase; ALT = Alanine aminotransferase; GGT = Gamma-Glutamyl-Transpeptidases; MCP-1 = Monocyte chemotactic protein-1; HOMA-IR = homeostatic model assessment; Adipo-IR = Adipose Tissue IR.

**Table 2:** Amino acids profile and its relationship with SVR and viral load.

Amino Acids mmol/L	Non Responders	Responders	NR vs R (*p* Value)	Viral Load (R)
Alanine	517.38 ± 25.4	508.10 ± 29.88	ns	ns
Phenylalanine	132.73 ± 7.65	129.78 ± 9.6	ns	
Glycine	287.4 ± 12.6	289.13 ± 15.62	ns	
Glutamate	455.5 ± 29.08	346.12 ± 33.52	0.006	R = 0.38
Histidine	131.75 ± 16.57	133.92 ± 8.12	ns	
Leucine	348.1 ± 34.73	323.98 ± 40.71	ns	
Isoleucine	83.68 ± 3.82	78.53 ± 5.41	ns	R = 0.25
Lysine	282.2 ± 22.08	262.76 ± 31.81	ns	R = 0.23
Proline	275.13 ± 21.44	213.46 ± 20.74	0.04	R = 0.34
Serine	259.5 ± 15.53	247.81 ± 20.47	ns	
Tyrosine	137 ± 8.58	120.27 ± 11.14	ns	R = 0.27
Valine	174.81 ± 8.9	169. ± 10.7	ns	
BCAAs	528.92 ± 31.25	496.7 ± 31.1	ns	R = 0.32
AAAs	258.26 ± 13.92	258.33 ± 21.17	ns	R = 0.32

## Abbreviations

CLD	Chronic Liver Disease
IR	Insulin Resistance
*DAA*	Directly Acting Antivirals-
SVR	Sustained Virological Response
PEG-IFNa	Pegylated-Interferon-A
RBV	Ribavirin
GC/MS	Gas Chromatography Mass Spectrometry
UHPLC/MS-QTOF	Ultra-High Performance Liquid Chromatography-Quadrupole Time-Of-Flight Mass Spectrometry
HOMA	Homeostasis Model Assessment
BCAA	Branched-Chain Amino Acids
AAA	Aromatic Amino Acids
SFA	Saturated Fatty Acids
Cer	Ceramides
PC	Phosphocholines
LysoPC	Lysophosphtidilcholines
MAG	Monoacylglycerols
DAG	Diacylglycerols
TAG	Triacylglycerols
SM	Sphingomyelin
